# Large neutral amino acid uptake and mTOR activation within CD4 T cells coordinate type 2 immunity and host resistance to *Trichuris muris*

**DOI:** 10.1093/discim/kyag003

**Published:** 2026-02-21

**Authors:** Maria Z Krauss, Kelly S Hayes, Suzanne H Hodge, Ana Villegas-Mendez, Matthew R Hepworth, Linda V Sinclair, Kevin N Couper, Richard K Grencis

**Affiliations:** Lydia Becker Institute of Immunology and Inflammation, School of Biological Sciences, Faculty of Biology, Medicine, and Health, University of Manchester, Manchester, UK; Lydia Becker Institute of Immunology and Inflammation, School of Biological Sciences, Faculty of Biology, Medicine, and Health, University of Manchester, Manchester, UK; Lydia Becker Institute of Immunology and Inflammation, School of Biological Sciences, Faculty of Biology, Medicine, and Health, University of Manchester, Manchester, UK; Cell Signalling and Immunology, University of Dundee, Dundee, UK; Lydia Becker Institute of Immunology and Inflammation, School of Biological Sciences, Faculty of Biology, Medicine, and Health, University of Manchester, Manchester, UK; Lydia Becker Institute of Immunology and Inflammation, School of Biological Sciences, Faculty of Biology, Medicine, and Health, University of Manchester, Manchester, UK; Cell Signalling and Immunology, University of Dundee, Dundee, UK; Lydia Becker Institute of Immunology and Inflammation, School of Biological Sciences, Faculty of Biology, Medicine, and Health, University of Manchester, Manchester, UK; Lydia Becker Institute of Immunology and Inflammation, School of Biological Sciences, Faculty of Biology, Medicine, and Health, University of Manchester, Manchester, UK; Manchester Cell-Matrix Centre, University of Manchester, Manchester, UK

**Keywords:** type 2 immunity, metabolism, host resistance, parasites, helminths

## Abstract

**Introduction:**

*Trichuris trichiura* (whipworm) is a gastrointestinal nematode that infects approximately 465 million people worldwide. *Trichuris muris* is used as a tractable model for the human whipworm. In wild-type mice, infection with a high dose of *T. muris* eggs leads to worm expulsion, which is dependent on a CD4Th2 response and interleukin (IL-)13 production. T cells up-regulate glycolysis and uptake of substrates following activation. The amino acid transporter SLC7A5 has been shown to be necessary for activation of mTORC1, a nutrient/energy/redox sensor critical for T cell differentiation into effector cells.

**Methods and Results:**

We found that mice lacking SLC7A5 in CD4T cells have significantly delayed worm expulsion, associated with reduced IL-13, reduced pmTOR, and reduced glycolytic rates. However, as infection progressed, IL-13 levels recovered in T cell-specific SLC7A5-deficient mice, alongside resistance. The critical role of CD4T cell metabolism *per se* and downstream mTOR in CD4T cells in host resistance was shown in mice lacking mTOR in CD4T cells that failed to expel their parasites and developed chronic infection.

**Conclusion:**

Our study shows that mTOR is essential for optimal functioning of T cells during whipworm infection and that deletion of Slc7a5 significantly delays worm clearance indicating a key role for amino acid acquisition by CD4T cells in resistance to helminth infection.

## Graphical Abstract

**Graphical Abstract:**
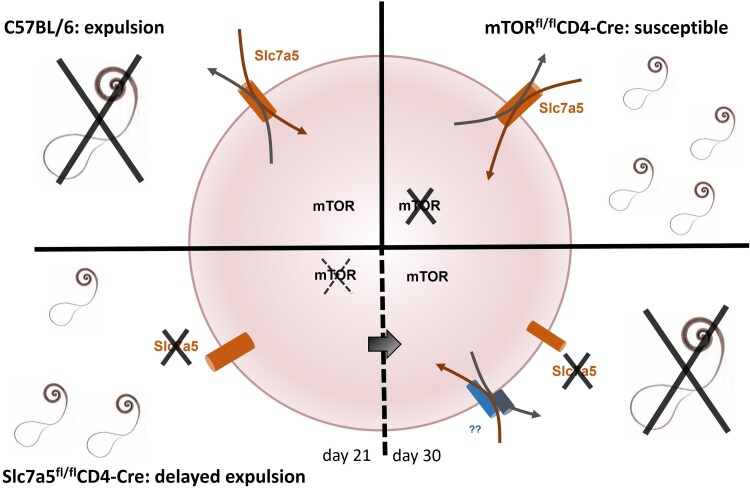

**Abbreviations:**
CD4T: CD4 positive T cell
ECAR: extracellular acidification rate
IL: Interleukin; ILC: innate lymphoid cell
LAT: large neutral amino acid transporter
LNAA: large neutral amino acid
mTOR: mechanistic target of rapamycin
mTORC: mechanistic target of rapamycin complex
MLN: mesenteric lymph node
OCR: oxygen consumption rate
SLC7A5: solute carrier family 7 member 5
STH: soil transmitted helminth
TCR: T cell receptor
Tfh: T follicular helper
Th: T helper

## Introduction

It is well established that cellular metabolism dictates the function of immune cells [1]. Remodelling metabolism is especially important for T cells. By trafficking through the body, they are exposed to different sites and nutrient availability and when activated they need to rapidly proliferate and secrete cytokines [2, 3]. The highly increased metabolic demand of activated T cells needs to be supported by increased uptake of nutrients such as lipids, glucose, and amino acids [4]. mTOR is multiprotein complex that plays a central role in an evolutionary conserved signalling pathway controlling cell growth and in T cells is a master regulator of metabolism that integrates activating cues received including *via* the TCR, co-stimulatory signals and cytokines, with signals from the environment [2, 5]. Recent work has shown that mTOR activity must be sustained, not just acutely activated, to maintain long-term T cell responses, particularly in chronic infection contexts [6]. mTOR then coordinates the activation of downstream signalling pathways that control nutrient uptake, glycolysis, mitochondrial biogenesis, and fatty acid oxidation [2]. Upon activation of naive CD4T cells, mTOR promotes upregulation of glycolysis [7]. mTOR^−^CD4T cells were shown to undergo normal activation but failed to differentiate into Th1, Th2, and Th17 subsets [8]. mTOR signalling occurs *via* two distinct complexes, mTORC1 and mTORC2 [9]. The main function of mTORC1 is to control cellular growth and modulate protein synthesis in T cells [5] with its action fine-tuned by the availability of nutrients such as amino acids and glucose [7] and the complex has been shown to be required for Th1 and Th17 differentiation [10, 11]. However, mTORC1 also plays critical roles in Th2 cell exit from quiescence and metabolic reprogramming [12]. mTORC2 regulates cellular functions such as actin reorganization and is important for Th2 cell differentiation [5, 10]. Following activation of CD4 T cells through the T cell antigen receptor, there is a marked increase in protein synthesis, which is required for the production of cytokines, which promote and regulate adaptive immunity [4, 5]. Recent studies have shown that the activation and differentiation of T cells are exquisitely dependent upon the uptake of amino acids through amino acid transporters, especially the large amino acid transporter SL7A5 [4, 5].

SLC7A5 (also known as large neutral amino acid transporter 1; LAT1) is the dominant large neutral amino acid transporter in activated T cells [4]. LAT1 has emerged as a critical metabolic checkpoint controlling T cell activation and has been identified as a potential therapeutic target in inflammatory diseases and cancer [13–15]. The importance of LAT family transporters extends to Group 2 innate lymphoid cells (ILC2s), which constitutively express high levels of both SLC7A5 (LAT1) and SLC7A8 (LAT2), with concurrent deletion of both transporters severely impairing ILC2 proliferation and cytokine production during helminth infection [16]. LAT1 is an antiporter that can transport several amino acids, with an important role to transport leucine and methionine, essential neutral amino acids [17]. Methionine is a critical amino acid used to initiate polypeptide and thus protein synthesis [6]. Intracellular leucine is required for mTOR activation by coordinating mTOR positioning on lysosomal membranes and RAG GTPase activity [18]. Methionine deprivation partially ablates mTOR activity in T cells [19]. Previous work has also shown the importance of SLC7A5 for Th1 and Th17 responses [4] although its role in Th2-controlled immunity is unclear. Indeed, our understanding of metabolism in type 2 immune responses has only begun to be elucidated. Recent advances have substantially improved our understanding of type 2 immune cell metabolism, demonstrating that Th2 cells and ILC2s rely on coordinated programmes of glycolysis, lipid metabolism, and amino acid uptake to support their effector functions [13, 20–22]. Multi-omics analyses have demonstrated that Th2 cell differentiation is accompanied by upregulation of glycolytic enzymes and nutrient transporters relative to naive CD4 T cells [20]. ILC2s, which initiate and amplify type 2 responses, exhibit dichotomous metabolic networks with distinct requirements for proliferation versus cytokine production [21, 22]. Amino acid availability acts as a metabolic rheostat for ILC2 responses, regulating mTOR-dependent proliferation and determining the magnitude of protective immunity during helminth infection [16]. Fatty acid metabolism, in addition to glucose utilization, plays critical roles in sustaining ILC2-mediated barrier protection during helminth infection and malnutrition [23]. Despite these advances, the specific metabolic requirements of Th2 cells during helminth infection and how metabolic constraints shape the efficacy of protective immunity remain incompletely understood.

Th2/type2 responses have co-evolved in order to mediate protective immune responses to large extracellular organisms, such as gastrointestinal helminths. Soil-transmitted helminths (STH), including *Ascaris lumbricoides* (roundworm), *Trichuris trichiura* (whipworm), *Necator americanus*, and *Ancylostoma duodenale* (hookworms) infect around 1.5 billion people worldwide [24]. Infections are concentrated in sub-Saharan Africa, Asia, and Latin America, in areas where sanitation is poor and malnourishment is frequent and are recognized as neglected tropical diseases [25]. Infections are acquired early in life, chronic infection is the norm through to adulthood with parasites placing significant metabolic demands on the host [26, 27]. *Trichuris muris* is a natural parasite of mice and is extensively utilized as a laboratory model for the study of human whipworm infection, *T*. *trichiura* [28], with remarkable similarity between species [29] and capacity to model both resistance and susceptibility. Resistance to *T. muris* is readily generated under experimental conditions and is dependent on a CD4 Th2 response and production of interleukin (IL-)13 [22, 23].

Here, we show that during acute *T. muris* infection the metabolic profile of CD4 T cells is altered to promote worm expulsion. Generation of protective immunity is associated with elevation of expression of nutrient transporters and increased glycolytic rates in the CD4T cell population. Using conditional knockout mice (*mTOR*^fl/fl^CD4-Cre mice), we show that T cell mTOR is essential for resistance against *T. muris*. Furthermore, deletion of *Slc7a5* in CD4 T cells reduces mTOR activity and significantly delays the generation of protective immunity and worm expulsion. Taken together, the data supports the hypothesis that CD4Th2 cells are sensitive in terms of their metabolic demands (in this case amino acids) during intestinal helminth infection and changes in their capacity to respond appropriately can significantly alter their ability to clear pathogens. This has implications not only for other intestinal helminth infections but also for the wider field of Th2-mediated immunological disease at mucosal surfaces.

## Results

When infected with a high dose of *T. muris* eggs, WT C57BL/6 mice develop a type2/Th2 response, which expels the parasites through increasing epithelial cell turnover, muscle contractility and mucus production [30–32]. Effective resistance to *T. muris* is dependent upon the generation of a strong and rapid CD4 T cell IL-13 response to expel the worm burden before immunomodulation by the parasite and chronic infection become established [33].

### CD4 T cells change their metabolism upon *T. muris* infection

**Figure 1:**
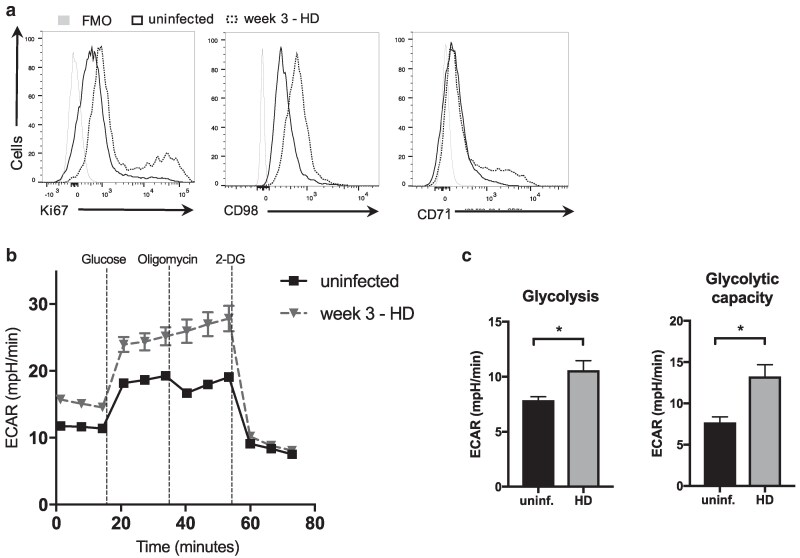
*Trichuris muris* infection alters expression of nutrient transporters and cell metabolism in CD4 T cells. (**a**) Flow cytometric analysis of Ki67, CD98, and CD71 in CD4 T cells from the mesenteric lymph node after 21 days of high-dose *T. muris* infection in C57BL/6 mice. (**b**) Extracellular flux analysis of CD4 T cells from MLN of high dose-infected and naive C57BL/6 mice. (**c**) Glycolytic rate and glycolytic capacity of CD4 T cells from MLN of naive and week 3 infected C57BL/6 were calculated following Seahorse’s guidelines. Glycolysis was determined as the maximum measurement before oligomycin injection minus the last measurement before glucose injection; glycolytic capacity was calculated as the maximum ECAR after oligomycin injection minus the last ECAR measurement before glucose injection. Data are representative of two independent experiments with five mice per group.

To assess the metabolic changes in CD4 T cells upon *T. muris* infection, C57BL/6 mice were infected with a high dose of infective eggs. CD71, the transferrin receptor was previously shown to be upregulated in activated T cells [34, 35], as well as CD98, which supports T cell proliferation by complexing with large amino acid transporters [36]. Three weeks post infection, the peak time of cytokine production in this system, CD4 T cells from the mesenteric lymph node (MLN) showed increased expression of the metabolism-related markers CD98 and CD71 and the proliferation marker Ki67 (Fig. 1a). Extracellular flux analysis of CD4T cells isolated from MLN of infected mice showed up-regulated glycolytic metabolism in comparison to CD4T cells from uninfected mice (Fig. 1b). CD4 T cells up-regulated both glycolytic rates and glycolytic capacity upon infection (Fig. 1c).

### T cell mTOR is required for resistance against *T. muris*

**Figure 2:**
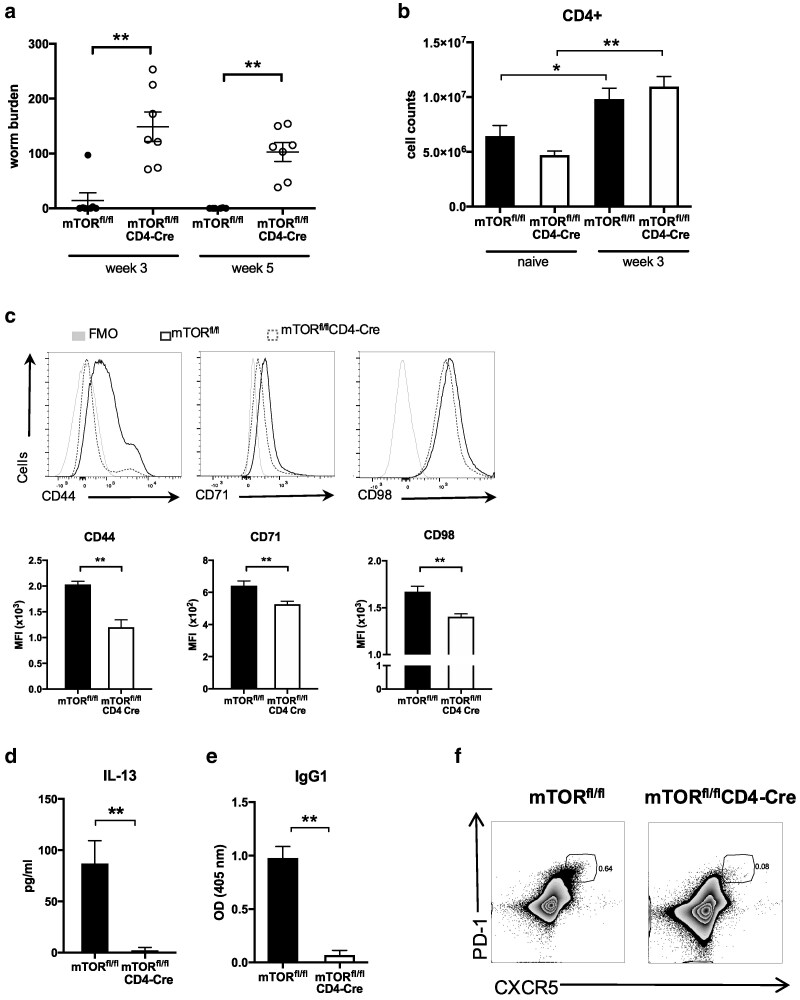
T cell mTOR is required for immunity to *T. muris*. (**a**) Worm burden from *mTOR*^fl/fl^ and *mTOR*^fl/fl^CD4-*Cre* mice after 3 and 5 weeks of high-dose *T. muris* infection. (**b**) Number of CD4 T cells in the MLN of naive and high-dose-infected mTOR^fl/fl^ and mTOR^fl/fl^CD4-Cre mice, analysed by flow cytometry. (**c**) Flow cytometry analysis of CD44, CD71 and CD98 in MLN cells from infected *mTOR*^fl/fl^ and *mTOR*^fl/fl^CD4-*Cre* mice. (**d**) MLN cells from *mTOR*^fl/fl^ and *mTOR*^fl/fl^CD4-*Cre* mice infected for 3 weeks were re-stimulated with parasite secretory/excretory product for 32 hours. Secretion of IL-13 was measured by cytometric bead array. (**e**) Levels of parasite-specific IgG1 in sera from week 3 infected *mTOR*^fl/fl^ and *mTOR*^fl/fl^CD4-*Cre* mice. (**f**) Flow cytometric analysis of follicular helper T cells (PD-1+ CXCR5+) from MLN of infected *mTOR*^fl/fl^ and *mTOR*^fl/fl^CD4-*Cre* mice. (a) Graph shows individual replicates of animals for two independent experiments combined (*n* = 3–4). (b–f) Data are representative of two independent experiments with 4–5 mice per group. * indicates *P* value ≤0.05, ** indicates *P* value ≤ 0.01 (unpaired *t* test).

**Figure 3:**
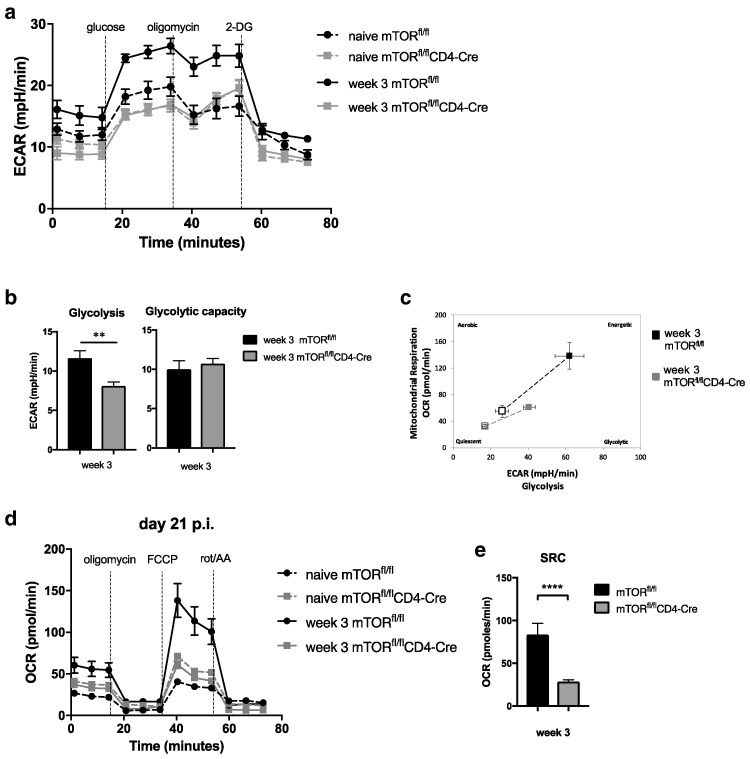
T cells fail to upregulate the glycolytic pathway and oxidative phosphorylation upon *T. muris* infection in the absence of mTOR. (**a**) CD4 T cells were isolated from MLNs of naive and week 3 infected *mTOR*^fl/fl^ and *mTOR*^fl/fl^CD4-*Cre* mice using magnetic labelling. CD4 T cells were subjected to a glycolytic stress test. (**b**) Quantification of glycolytic capacity and glycolysis levels of CD4 T cells from MLN of *mTOR*^fl/fl^ and *mTOR*^fl/fl^CD4-*Cre* mice obtained with extracellular flux analysis. (**c**) Cell energy phenotype from CD4 T cells isolated from MLN of infected *mTOR*^fl/fl^ and *mTOR*^fl/fl^CD4-*Cre* mice. Empty squares represent basal metabolic levels, and filled squares represent stressed measurements (after injection of oligomycin and FFCP). (**d**) Mitochondrial stress test from CD4 T cells from MLNs of week 3 infected mice. (**e**) Spare respiratory capacity of CD4 T cells from MLN of *mTOR*^fl/fl^ and *mTOR*^fl/fl^CD4-*Cre* mice obtained with extracellular flux analysis. Cells from each mouse were individually isolated and all groups were submitted to the extracellular flux analysis simultaneously to allow comparisons. Graphs show mean ±SEM and data are representative of two independent experiments with 3–4 mice per group. ** indicates *P* value ≤0.01, *** indicates *P* value ≤ 0.001 (Mann–Whitney test).

mTOR is a master regulator of T cell metabolism and in order to define its importance in host protection, *mTOR*^fl/fl^CD4-Cre mice and *mTOR*^fl/fl^ control mice were infected with a high dose of *T. muris* infection. *mTOR*^fl/fl^CD4-Cre mice failed to expel a high dose of *T. muris* infection, even after 5 weeks (Fig. 2a). *mTOR*^−^ CD4 T cells from MLN taken 3 weeks after infection (p.i.) showed lower expression of CD44 (T cell marker which is increased in expression upon T cell activation), CD71 and CD98 when compared to WT CD4 T cells at this time point (Fig. 2c). However, overall CD4 T cell counts in MLN from *mTOR*^fl/fl^CD4-Cre mice and mTOR^fl/fl^ mice were comparable (Fig. 2b). MLN cells from infected *mTOR*^fl/fl^CD4-Cre mice failed to produce IL-13 after re-stimulation with parasite E/S antigen (Fig. 2d). These cells, however, produced similar levels of other cytokines such as IFN-γ, IL-17A, and TNF-α when compared to cells from *mTOR*^fl/fl^ mice (Supplementary Fig. S1a). Parasite-specific IgG1 is detected in serum of wild-type (WT) mice in response to *T. muris* infection [37]. Serum levels of parasite-specific IgG1 found in infected *mTOR*^fl/fl^CD4-Cre mice were significantly reduced as compared to the levels found in infected control *mTOR*^fl/fl^ mice (Fig. 2e). Levels of parasite-specific IgM, however, were found at similar levels in sera from infected *mTOR*^fl/fl^CD4-Cre mice and *mTOR*^fl/fl^ mice, indicating a potential failure of T cells to support B cell class switching or to produce the appropriate class switching cues (Supplementary Fig. S1b). To investigate further the impairment of IgG1 observed when mTOR was deleted in T cells, we also assessed the follicular helper T cell population upon infection in *mTOR*^fl/fl^CD4-Cre and mTOR^fl/fl^ mice. The PD-1+ CXCR5+ Tfh population was absent in infected *mTOR*^fl/fl^CD4-Cre mice (Fig. 2f). Expression of Bcl-6, required for Tfh cell differentiation, was also found reduced in CD4 T cells from these mice (Supplementary Fig. S2). This confirms previous reports showing that mTOR is required for antibody class switching and Tfh differentiation [38, 39]. We next assessed the impact of the deletion of mTOR on the metabolism of CD4 T cells during infection, using extracellular flux analysis. *mTOR*^−^ CD4 T cells from MLN not only failed to up-regulate glycolysis upon infection (Fig. 3a and b), but also showed lower levels of mitochondrial respiration when compared to WT CD4 T cells (Fig. 3c and e). The metabolic phenotype of *mTOR^−^* CD4 T cells from MLN of infected mice was more quiescent-like than WT CD4 T cells from infected mice (Fig. 3c). We assessed caecal crypt length to determine intestinal pathology on *mTOR*^fl/fl^CD4-Cre and *mTOR*^fl/fl^ mice and despite having very high worm burdens for long periods of time, *mTOR*^fl/fl^CD4-Cre mice did not present with any aberrant pathologies (Supplementary Fig. S3).

### Deletion of *Slc7a5* in T cells leads to impairment of the Th2 response 3 weeks post infection

**Figure 4:**
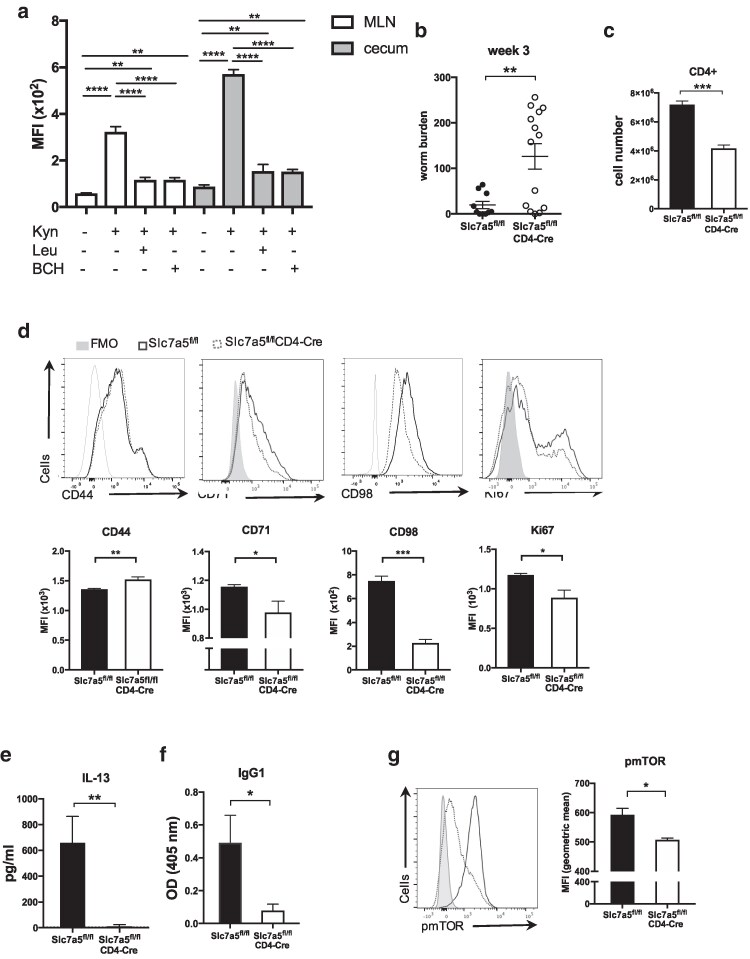
CD4 T cells transport kynurenine *via* the system L transporter SLC7A5 during *T. muris* infection and *Slc7a5*^fl/fl^CD4-*Cre* mice exhibit impaired Th2 response 3 weeks after high-dose *Trichuris muris* infection compared with wild-type (*Slc7a5*^fl/fl^) mice. (**a**) Kynurenine (200 μM) uptake in CD4 T cells from week 3 high dose-infected C57BL/6 mice in the presence or absence of leucine (5 mM) and BCH (10 mM). (**b**) Number of *T. muris* larvae found in the caecum and proximal colon of *Slc7a5*^fl/fl^CD4-*Cre* and wild-type mice 3 weeks after high-dose infection. (**c**) Numbers of CD4 T cells in MLN of *Slc7a5*^fl/fl^CD4-*Cre* mice and littermate controls at 3 weeks p.i. (**d**) Flow cytometric analysis of CD44 in CD4 T cells and of CD71, CD98, and Ki67 in CD44hi CD4 T cells in MLN from infected *Slc7a5*^fl/fl^ and *Slc7a5*^fl/fl^CD4-*Cre* mice on week 3 p.i. (**e**) MLN cells from infected *Slc7a5*^fl/fl^ and *Slc7a5*^fl/fl^CD4-*Cre* mice were re-stimulated with parasite excretory/secretory product for 32 hours and IL-13 levels in cell supernatant were assessed by cytometric bead array. (**f**) Serum levels of parasite-specific IgG1 in *Slc7a5*^fl/fl^ and *Slc7a5*^fl/fl^CD4-*Cre* mice 3 weeks after *T. muris* infection. (**g**) *Ex vivo* flow cytometric analysis of phosphorylated mTOR in CD4 T cells from MLN of infected *Slc7a5*^fl/fl^ and *Slc7a5*^fl/fl^CD4-*Cre* mice on week 3 p.i. Graphs show mean ±SEM and data are representative of 2–3 independent experiments with 3–5 mice per group. * indicates *P* value ≤0.05, ** indicates *P* value ≤ 0.01, ***indicates *P* value ≤ 0.001 (unpaired *t*-test).

We questioned the nutrient transport systems that were required for mTOR activation and CD4 T cell metabolic reprogramming during *T. muris* infection. Previous studies have shown that SLC7A5 is required for T cell differentiation and clonal expansion, e.g. for transporting leucine that is required inside the cell for mTORC activation and for sustaining c-Myc expression [4, 18, 40]. To assess the uptake of large neutral amino acids (LNAAs) by CD4T cells following *T. muris* infection, kynurenine uptake was assessed *ex vivo*. Kynurenine uptake is an accurate proxy to measure LNAA uptake, and uptake has been shown to be SLC7A5 dependent in murine T cells [41]. The data shows that following infection, activated CD4 T cells in both MLN and caecum utilize SLC7A5 to transport amino acids, and the addition of excess leucine competitively blocks the uptake of kynurenine to comparable levels seen using the system L blocker BCH (Fig. 4a). To assess the importance of SLC7A5 for a type 2 response, *Slc7a5*^fl/fl^CD4-*Cre* mice [4] and *Slc7a5*^fl/fl^ control mice received a high-dose infection of *T. muris*. We assessed infection of *Slc7a5*^fl/fl^CD4-*Cre* mice and in their littermate controls after 3 and 5 weeks to measure the peak cytokine responses and progression of the infection to patency, respectively. *Slc7a5*^fl/fl^CD4-*Cre* mice showed significantly higher worm burdens than *Slc7a5*^fl/fl^ control mice 3 weeks after a high-dose infection of *T. muris* (Fig. 4b). *Slc7a5*^fl/fl^CD4-*Cre* mice also showed reduced numbers of CD4 T cells in the MLN (Fig. 4c). Frequencies of the activation marker CD44 in CD4 T cells from MLNs of *Slc7a5*^fl/fl^CD4-*Cre* mice were slightly higher than those found in *Slc7a5*^fl/fl^ mice at week 3 p.i. However, percentages of CD71 and CD98 and the proliferation marker Ki67 in these CD44hi CD4 T cells were found to be lower in the absence of T cell *Slc7a5* at 3 weeks p.i. (Fig. 4d). Re-stimulation of MLN cells from infected mice with parasite secreted/excreted (E/S) antigen revealed that production of IL-13, the key cytokine required for worm expulsion [22] was abrogated in *Slc7a5*^fl/fl^CD4-*Cre* mice (Fig. 4e). Serum levels of parasite-specific IgG1 were much lower in infected *Slc7a5*^fl/fl^CD4-*Cre* mice when compared to levels found in infected *Slc7a5^f^*^l/fl^ control mice (Fig. 4f). CD4 T cells from MLNs of *Slc7a5*^fl/fl^CD4-*Cre* mice showed lower phosphorylation of mTOR (S2448) than CD4 T cells from MLNs of infected *Slc7a5*^fl/fl^ control mice (Fig. 4g).

### Worm expulsion and IL-13 production are partially rescued in *Slc7a5*^fl/fl^CD4-*Cre* mice at later stages of infection

**Figure 5:**
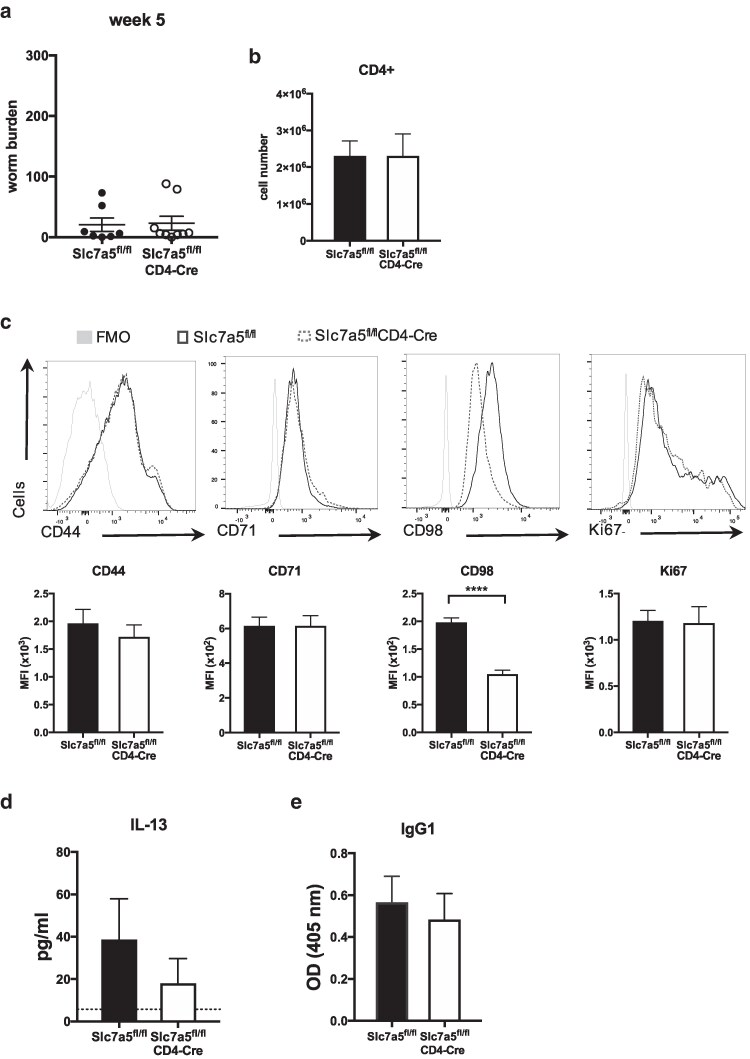
CD4 T cells overcome the lack of SLC7A5 at the later stage of *T. muris* infection. (**a**) Worm burden after 5 weeks of high-dose *T. muris* infection in *Slc7a5*^fl/fl^ and *Slc7a5*^fl/fl^CD4-*Cre* mice. (**b**) Numbers of CD4 T cells in MLN after 5 weeks of high-dose infection. (**c**) Flow cytometry analysis of CD44 in CD4 T cells and of CD71, CD98, and Ki67 in CD44hi CD4 T cells in MLN CD4T cells from 5-week-infected *Slc7a5*^fl/fl^ and *Slc7a5*^fl/fl^CD4-*Cre* mice. (**d**) MLN cells from infected *Slc7a5*^fl/fl^ and *Slc7a5*^fl/fl^CD4-*Cre* mice were incubated with the excretory/secretory antigen from the parasite for 32 hours. Levels of secreted IL-13 in cell supernatants were measured by cytokine bead array. Dotted line represents IL-13 secretion by cells from uninfected mice. (**e**) Serum levels of parasite-specific IgG1 from 5-week-infected *Slc7a5*^fl/fl^ and *Slc7a5*^fl/fl^CD4-*Cre* mice. (a) Graph shows individual replicates of animals for two independent experiments combined (*n* = 4–5). (b–e) Graphs show mean ± SEM and data are representative of two independent experiments with 4–5 mice per group.

When assessed after 5 weeks of a high-dose *T. muris* infection, *Slc7a5*^fl/fl^CD4-*Cre* mice showed similar worm burdens to *Slc7a5*^fl/fl^ control mice, with most of the animals having expelled infection (Fig. 5a). At this time point, MLNs from *Slc7a5*^fl/fl^CD4-*Cre* mice had comparable numbers of CD4 T cells to those from *Slc7a5*^fl/fl^ mice (Fig. 5b). Frequencies of CD44, CD71, and Ki67 CD4 T cells were also found to be similar in MLN from infected *Slc7a5*^fl/fl^CD4-*Cre* and infected *Slc7a5*^fl/fl^ mice. Expression of CD98, however, remained much lower in CD4 T cells from MLN of *Slc7a5*^fl/fl^CD4-*Cre* after 5 weeks of infection (Fig. 5c). RT-PCR *Slc7a5* analysis in CD4 T cells ruled out the possibility that *Slc7a5*^fl/fl^CD4-*Cre* mice started to re-express *Slc7a5* later stages of infection (Supplementary Fig. S4), although RT-PCR *Slc7a8* analysis suggested there was a modest recovery in expression of *Slc7a8* in CD4 T cells later in infection (Supplementary Fig. S4). Production of IL-13 by MLN cells upon re-stimulation with parasite E/S antigen was found to be similar between *Slc7a5*^fl/fl^CD4-*Cre* and *Slc7a5*^fl/fl^ mice after 5 weeks of infection (Fig. 5d). Also, serum levels of parasite-specific IgG1 were no longer different between both groups at this time point (Fig. 5e). However, CD4 T cells from *Slc7a5*^fl/fl^CD4-*Cre* mice continued to exhibit impaired kynurenine uptake 5 weeks post infection (Supplementary Fig. S5). Data from this later time point demonstrated that deletion of *Slc7a5* in T cells significantly delays worm expulsion and the type 2 response but does not induce complete susceptibility to a high-dose infection of *T. muris.* Interestingly, following the delayed expulsion of the parasites, *Slc7a5*^fl/fl^CD4-*Cre* mice can subsequently expel *T. muris* as fast as *Slc7a5*^fl/fl^ mice when given a secondary challenge infection (Supplementary Fig. S6).

### CD4 T cells that lack *Slc7a5* show lower glycolytic rates at the earlier stage of *T. muris* infection

**Figure 6:**
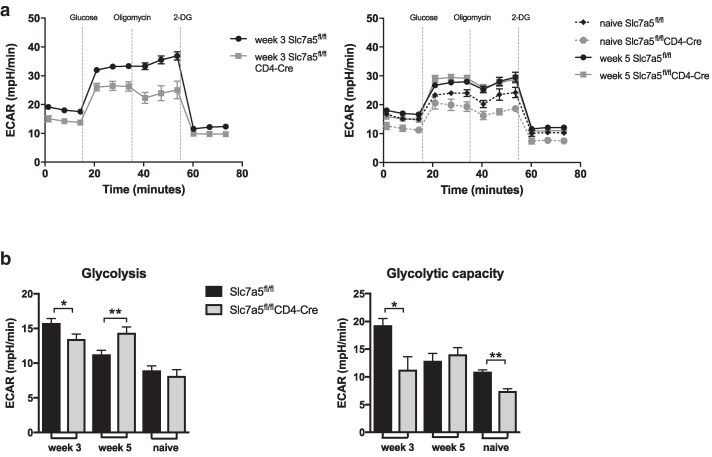
CD4 T cells lacking SLC7A5 show impaired glucose metabolism 3 and 5 weeks post *T. muris* infection. (**a**) CD4 T cells from MLNs of infected and naive *Slc7a5*^fl/fl^ and *Slc7a5*^fl/fl^CD4-*Cre* mice were isolated using magnetic beads and subjected to a glycolytic stress test. (**b**) Shows glycolytic levels and glycolytic capacity of CD4 T cells from MLNs of *Slc7a5*^fl/fl^ and *Slc7a5*^fl/fl^CD4-*Cre* mice obtained with extracellular flux analysis. Cells from each mouse were individually isolated and all groups were submitted to the extracellular flux analysis simultaneously to allow comparisons across time points. Graphs show mean ± SEM and data are representative of two independent experiments with 3–4 mice per group. * indicates *P* value ≤0.05, ** indicates *P* value ≤ 0.01 (Mann–Whitney test).

Previous studies have shown that SLC7A5 was required for mTOR activation and expression of glucose transporters in T cells and, consequently, to support glycolysis [17, 18]. Using extracellular flux analysis, we investigated if deletion of *Slc7a5* in CD4 T cells from MLN resulted in impaired metabolic shift during infection. CD4 T cells were isolated from MLN of infected *Slc7a5*^fl/fl^CD4-*Cre* and infected *Slc7a5*^fl/fl^ mice and subjected to a glycolytic stress test. At week 3 p.i., CD4 T cells from *Slc7a5*^fl/fl^CD4-*Cre* mice showed lower glycolysis and glycolytic capacity when compared to those from littermate control mice (Fig. 6a). Levels of glycolytic capacity were also lower in CD4 T cells from MLNs of naive *Slc7a5*^fl/fl^CD4-*Cre* mice when compared to those from naive littermate control mice. Notably, however, after 5 weeks of *T. muris* high-dose infection, SLA7A5^−^ CD4 T cells from MLNs showed similar glycolytic capacity and higher glycolytic levels to CD4 T cells from MLNs of littermate control mice (Fig. 6b). We, therefore, confirmed that deletion of *Slc7a5* in T cells impacts T cell activation and metabolism during early but not late stages of infection.

## Discussion

In this study we have demonstrated that deletion of the amino acid transporter SLC7A5 expressed by CD4 T cells significantly delays expulsion of a high-dose *T. muris* infection and also that CD4 T cell mTOR is required for host protective immunity. Assessment of the immune response at 3 weeks post infection revealed impairment of T cell function upon deletion of SLC7A5 in T cells, which included abrogation of IL-13 and parasite-specific IgG production. SLC7A5 transports a number of essential amino acids including leucine, which has been shown to be essential for mTOR activation [42, 43]. Indeed, phosphorylation of mTOR was found to be decreased in SLC7A5-deficient CD4 T cells at 3 weeks post infection, suggesting that a lack of intracellular amino acids required for mTOR activation may be responsible. Also, at this time point, CD4 T cells lacking SLC7A5 exhibited a muted up-regulation of glycolysis compared to wild type (WT) CD4 T cells. *Slc7a5*^fl/fl^CD4-*Cre* mice showed significantly reduced production of parasite-specific IgG at 3 weeks post infection, similarly to that observed in *mTOR*^fl/fl^CD4-*Cre* mice suggesting that SLC7A5 could be important for T follicular helper cell function. At later time points, however, *Slc7a5*^fl/fl^CD4-*Cre* mice eventually generated an immune response which, although restricted, was sufficient to promote worm clearance. The possibility that *Slc7a5*^fl/fl^CD4-*Cre* mice started to re-express Slc7a5 was ruled out by RT-PCR *Slc7a5* analysis in CD4 T cells from the later stages of infection. This suggests compensatory mechanisms came into play or a temporal importance of SLC7A5 on T cells during infection. CD98 can form heterodimers with SLC7A6, SLC7A7, or SLC7A8 to form the y + LAT2, y + LAT1, or LAT2 transport systems, respectively, that transport leucine and other large amino acids [36]. In this case, given that expression of CD98 remained low during the later phase, this would suggest that the compensatory mechanism was at least partially independent of CD98/LAT systems. It was, however, interesting to note the modest recovery in CD4 T cell expression of *Slc7a8* on day 34 post infection especially when taken together with the recent description of an important role for SLC7A8 in Th2 cells [44]. We have previously demonstrated that deletion of SLC7A5 in ILC2s also led to lower surface expression of CD98 and did not increase expression of SLC7A8 [16]. It is also possible other amino acid transporters may be involved or indeed glucose transporters, which may boost the protein synthesis required to generate protective CD4 T cells. After the compensatory mechanisms are in place, the cell is functional and, interestingly, *Slc7a5*^fl/fl^CD4-*Cre* mice can subsequently expel *T. muris* as fast as *Slc7a5*^fl/fl^ mice when re-challenged perhaps suggesting that once an effective Th2 response has been primed, the absence of SLC7A5 is not critical for anamnestic immunity (Supplementary Fig. S6). Thus, early nutrient sensing by T cells together with the availability of amino acids and glucose are key to the generation of efficient responses to *T. muris*. Moreover, the increases in expression of CD98 and CD71 by CD4 T cells after infection could reflect other additional metabolic changes operating over and above their association with T cell activation, such as roles in stabilizing glucose transporters [45] or uptake of iron (CD71), important for mitochondrial respiration [46].

It is noteworthy that mice fed a low-protein diet showed impaired resistance to a high dose of *T. muris* infection [47]. Intestinal nematode infection has multiple effects upon the metabolic phenotype of the host tissue [27] and nematodes themselves require the same resources for development and rapid growth. Indeed, transcriptomic analysis of *T. muris* confirms expression of parasite amino acid transporter genes including a large amino acid transporter and parasite glucose transporter genes in the larval stages that are present during the first 21 days of infection [29]. Moreover, the bacillary band, a modified region of the cuticle found in *T. muris* and other parasitic nematodes that occupy an intestinal epithelial niche, has also been shown to readily absorb glucose from the environment [48]. Taken together, it is reasonable to suggest the parasites themselves are competing with host tissue, including T cells, for these valuable resources during the establishment of infection.

We also demonstrate that mTOR plays a major role in T cell- mediated immunity against *T. muris*. The deletion of mTOR in T cells had a severe impact on the immune response to *T. muris* and made mice completely susceptible to high-dose infections. *mTOR*^fl/fl^CD4-*Cre* mice showed abrogated production of IL-13, impaired Tfh differentiation and antibody class switching. As shown by data from the extracellular flux analysis, mTOR appears to not only be required for upregulation of aerobic glycolysis in T cells but also for increased mitochondrial respiration during *T. muris* infection. This is in line with previous studies using other cell types that showed that mTOR can also promote mitochondrial activity [49, 50]. The role of mTOR during intestinal infection has received attention with T cell mTOR essential for resistance to *Citrobacter rodentium* infection in mice [51], although in this case, for the generation of efficient host protective Th1 responses in the intestinal mucosa. Studies of the protozoan parasite, *T. muris* added a further level of complexity showing that intestinal epithelial cell-specific mTORC1 was important in the initiation of Type 2 immune responses [52], whereas it has previously been shown that mTORC2 promoted Th2 responses [10].

Increased glycolytic levels were observed in CD4 T cells upon infection with *T. muris* and support a role for glycolysis in protective immunity. This is in line with several previous studies which have described aerobic glycolysis as a requirement for cytokine production in T cells [53, 54]. Tibbit *et al* showed that Th2 cells are heavily reliant on glycolysis for their differentiation and function and that blocking glycolysis *in vivo* using 2-DG impairs the production of IL-13 by Th2 cells [55]. Our work confirms the correlation between glycolysis and IL-13 production and reinforces that upregulation of glycolysis is dependent on mTOR. Overall, data presented here have demonstrated that CD4 T cell mTOR acts as major regulator of the protective immune response to *T. muris*. Deletion of mTOR in T cells results in failure to upregulate metabolic pathways, which leads to major impairment of T cell activation and function and therefore susceptibility to *T. muris*. Moreover, deletion of SLC7A5 in T cells leads to considerable impairment of the effector function of T cells in the early stage of infection. This reinforces that amino acid transport and availability sensing are key steps in licensing mTOR activation. T cells lacking SLC7A5, however, partially recover function later on infection and promote parasite expulsion. The finding that alteration of a single amino acid transporter could significantly influence the generation of protective immunity to an intestinal helminth confirms the importance of immune cell metabolism following infection (Supplementary Fig. S7, *Graphical Abstract*). For *T. muris* infection, the generation of a rapid protective Th2 response is key to resistance. A delay in worm expulsion allows the parasite to establish a non-protective Th1 response and chronic infection, which impacts on long-term resistance/susceptibility [28, 29, 33]. The present work demonstrates the fragile equilibrium of nutrient availability and nutrient transporters that exists for efficient T cell effector function at mucosal sites and it is intriguing to speculate that individuals with mutations in amino acid transporters that affect efficient transport may be compromised in their capacity to generate rapid CD4 T cell IL-13 responses. In endemic areas of gastrointestinal nematode infection, infected individuals are often malnourished. It is reasonable to speculate, for example, that low-protein diets could contribute to making hosts more susceptible to helminths and potentially to other kinds of infection, due to compromised metabolic activity of mucosal immune T cells.

## Methods and materials

### Animals

*Slc7a5* ^fl/fl^CD4-*Cre* mice were obtained from Prof. Doreen Cantrell (University of Dundee, UK) [4]. *mTOR* mice were originally purchased from JAX (USA) and crossed with CD4-*cre* mice [56]. For both strains knockout (KO) and corresponding WT littermate controls were bred in-house at the Biological Services Facility (BSF) at the University of Manchester. Experiments were performed under the regulations of the Home Office Scientific Procedures Act (1986) (Licence P043A082) and were subject to local ethical review by the University of Manchester Animal Welfare and Ethical Review Body (AWERB) and followed ARRIVE 2.0 guidelines. Animals were maintained in individually ventilated cages with 65% humidity at 21°C–23°C. Mice were used at 6–10 weeks of age and infected and naive mice were co-housed. Mice were sex- and age-matched for experiments. The mice utilized in these experiments were not randomized but cages were randomly assigned to different treatment groups.

### Infections and worm counts

Parasite maintenance and extraction of the E/S antigen were performed as described previously [57]. For high-dose infections, mice were infected with approximately 300 infective *T. muris* eggs in ddH_2_O via oral gavage. For low-dose infections, mice were given 20 infective eggs in ddH_2_O via oral gavage. Worm burdens were assessed at 21 or 32–35 days post infection. For worm burden determination, the caecum and proximal colon were removed and cut longitudinally in a Petri dish with water. The intestinal contents were removed, the epithelium was scraped using curved forceps and the worms were individually removed and counted under a dissecting microscope.

### Cell culture and cytokine analysis

MLN cells were collected and re-stimulated for 32 hours as previously described [58]. Secreted concentrations of IL-13 and interferon (IFN)-γ were measured from cell supernatant using a cytometric bead array (CBA, BD Biosciences, UK) according to the manufacturer’s instructions.

### Isolation of lamina propria lymphocytes

Caeca and the proximal colon were collected at autopsy individually into complete RPMI 1640 medium. Adipose tissue was removed, intestines were sectioned length wise and washed 3 times in HBSS 2% FCS. Intestines were snipped into small sections (∼0.5 cm), washed in HBSS without FCS and incubated with HBSS EDTA (2 mM) for 10 minutes at 37°C in a tube rotator. Tissue snips were incubated in HBSS EDTA again for another 30 minutes. Gut tissue was then washed in PBS and digested for 30 minutes at 37°C using 10 ml of complete RPMI containing the following enzymes: collagenase V (0.85 mg/ml), collagenase D (1.25 mg/ml), dispase (1 mg/ml), and DNase (30 μg/ml), all Sigma. Tubes were kept in the rotor for all the incubation time and lamina propria lymphocytes were passed through a 70 μm strainer to remove any tissue debris.

### Parasite-specific antibody analysis

Serum levels of parasite-specific antibodies were measured by ELISA as described previously [57].

### Flow cytometry

Flow cytometry experiments were performed on a BD LSRII or Fortessa and analysed using FlowJo software. The following antibodies were used for flow cytometry: anti-CD4 (GK1.5, Biolegend), anti-CD98 (RL388, Biolegend), anti-PD-1 (29F.1A12, Biolegend), anti-CD71 (R17217, Biolegend), anti-Ki67 (B56, eBioscience), CXCR5 (L138D7, Biolegend), anti-pmTOR (MRRBY, eBioscience), anti-CD44 (IM7, Biolegend). Intracellular staining was performed using Fixing/Permeabilization Foxp3 kit (eBioscience). For assessment of phosphorylated mTOR, cells were fixed with 4% paraformaldehyde and permeabilized with methanol. Kynurenine uptake assays were performed as described previously [41].

### Extracellular flux analysis (Seahorse)

CD4 T cells were isolated from MLNs using magnetic positive selection (L3T4 beads, Miltenyi) according to the manufacturer’s instructions. Cellular metabolism was assessed using a Seahorse Bioscience XF96e Extracellular Flux Analyzer. Approximately 200 000 isolated CD4 T cells were adhered to the Seahorse plate using Cell-Tak (Corning) and cultured in Seahorse media for 30 minutes to 1 hour. ECAR was measured in XF media (modified DMEM containing 2 mM L-glutamine) under basal conditions, in response to 10 mM glucose, 2 μM oligomycin, and 100 mM 2-DG. OCR was measured under basal conditions after injection of 2 μM oligomycin, 1.5 μM FCCP, and 0.5 μM rotenone + 0.5 μM antimycin A (all Sigma). If necessary, data was normalized by cell number after analysis.

### Kynurenine uptake assay

Following tissue processing, single-cell suspensions were stained for extracellular antibodies. After staining incubations, tubes were centrifuged at 1500 rpm for 5 minutes, washed once with and Hank’s Balanced Salt Solution (HBSS), centrifuged again and resuspended in 200 µl per sample of HBSS. To each sample, either HBSS, 5 nM lysine (Sigma), 5 nM leucine, or 10 mM BCH was added, followed by 200 µM kynurenine (Sigma) to all samples, except negative control samples, which received HBSS alone. After 5 minutes (at 37°C) 125 µl of 4% PFA or 100 μl BD Cytofix (BD Biosciences) was added and left for 20 minutes at room temperature. Tubes were then spun (as above) and resuspended in 250 µl of HBSS before analysis with flow cytometry, no longer than 45 minutes after assay was finished. Some experiments also included samples that were incubated with 5 nM alanine (Sigma) or 5 nM methionine (Sigma), followed by 200 µM kynurenine and incubated as described above.

### Statistical analysis

All statistical analysis was performed using GraphPad Prism (GraphPad Software). Data distribution was tested for normality using the Shapiro–Wilk test. Comparisons between two groups were carried out using an unpaired *t* test (for parametric data) or a Mann–Whitney test (for non-parametric data).

## Supplementary Material

kyag003_Supplementary_Data

## Data Availability

The data underlying this article will be shared on reasonable request to the corresponding author.
