# Supplementary material for: Large neutral amino acid uptake and mTOR activation within CD4 T cells coordinate type 2 immunity and host resistance to Trichuris muris
Source: Discov Immunol. 2026 Feb 21;5(1):kyag003. doi: 10.1093/discim/kyag003 (PMC12971014; doi:10.1093/discim/kyag003)
Supplement: kyag003_Supplementary_Data [file kyag003_supplementary_data.docx]

**SUPPLEMENTARY FIGURES**


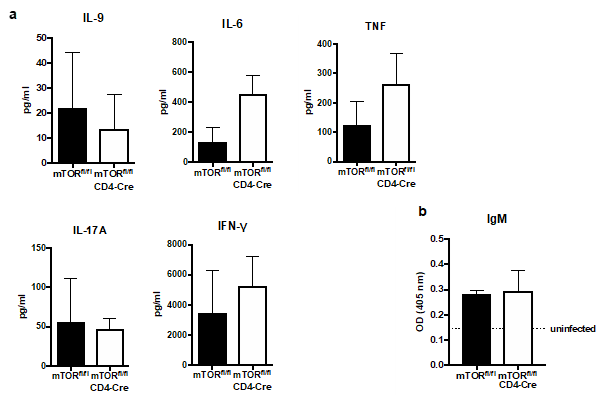


**Fig. S1. Cytokine and antibody production in infected *mTOR*^fl/fl^ and *mTOR*^fl/fl^CD4-*Cre*.** (a) MLN cells obtained from *mTOR*^fl/fl^ and *mTOR*^fl/fl^CD4-*Cre* mice infected for 3 weeks were re-stimulated *in vitro* with parasite secretory/excretory product for 32 hours. Secretion of cytokines was measured by cytometric bead array. (b) Serum levels of parasite-specific IgM in *mTOR*^fl/fl^ and *mTOR*^fl/fl^CD4-Cre mice after 30 days of infection**.** Mice received 300 infective eggs. *T. muris* E/S was used as antigen. Bar chart shows values for IgM at a 1:20 dilution. Values represent means +/- SEM (n=3).

**Fig. S2. Bcl6 in CD4+ T cells from MLNs of *T. muris* infected *mTOR*^fl/fl^ and *mTOR*^fl/fl^CD4-*Cre* mice**. MLNs were collected from mice at day 35 post *T. muris* infection with 300 eggs. n=3. Bcl6 was detected by flow cytometry.

**Fig. S3. Caecal crypt length in naïve and *T. muris* infected *mTOR*^fl/fl^ and *mTOR*^fl/fl^CD4-Cre mice**. Mice were culled after 21 or 35 days of receiving a *T. muris* high dose infection. Periodic Acid Schiff staimning. Photographs at x10 magnification were analysed using ImageJ software. Arrows indicate a *T. muris* in cross section. Bars indicate mean (n=5-8). * indicates p value≤ 0.05, **indicates p value≤ 0.01, ***indicates p value≤ 0.001.


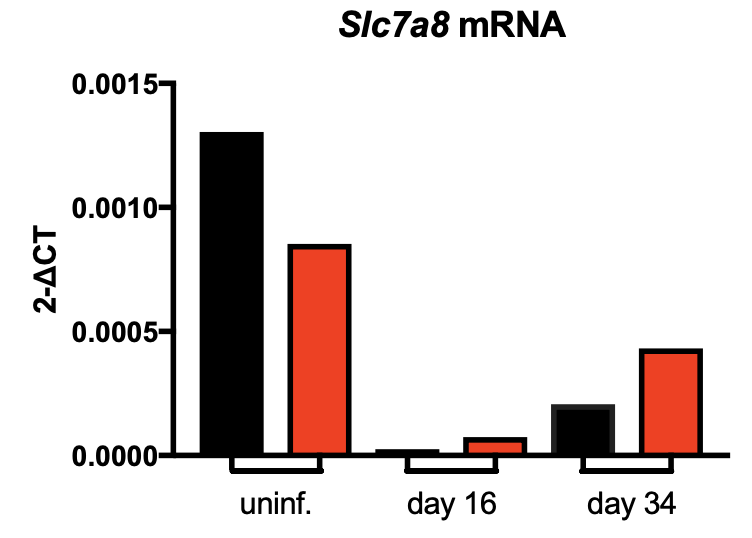


**Fig. S4. Detection of *Slc7a5* or *Slc7a8* in CD4 T cells from *Slc7a5*^fl/fl^ and *Slc7a5*^fl/fl^CD4-*Cre* mice by RT-qPCR.** MLNs were collected after 16 or 34 days of a high dose (300 eggs) *T. muris* infection and from uninfected mice. Cells from 3-5 mice were pooled. Values represent mean CT normalised by β-actin.


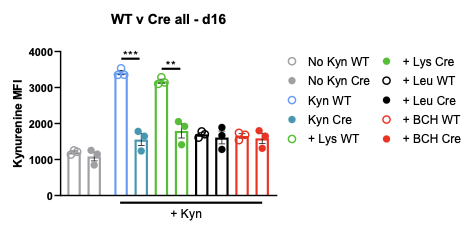


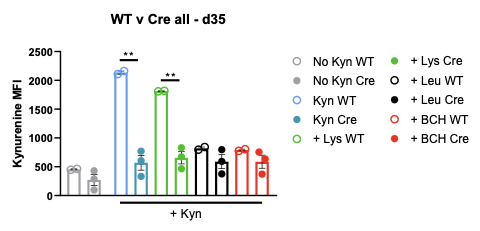


**Fig. S5 Kynurenine uptake in CD4 T cells of *Slc7a5*^fl/fl^ (WT) and *Slc7a5*^fl/fl^CD4-*Cre* (Cre) mice at day 16 and day 35 post infection.** (a) Kynurenine (200μM) uptake in CD4+ T cells from MLNs were assessed by flow cytometry in presence of leucine (5mM), lysine (5mM) or the system LAA transporter inhibitor BCH (10mM). All data was normalised to the lowest value as no kyn controls were consistently less than 0. n=2-3

**Fig. S6. Worm burden of *Slc7a5*^fl/fl^ and *Slc7a5*^fl/fl^CD4-*Cre* mice upon *T. muris* high dose re-challenge.** Mice received a high dose of 300 eggs. At day 26 p.i. animals received a second high dose infection (300 eggs). Worm burdens were assessed at day 20 p.i. n=3.

**Fig. S7. Graphical Abstract**

**
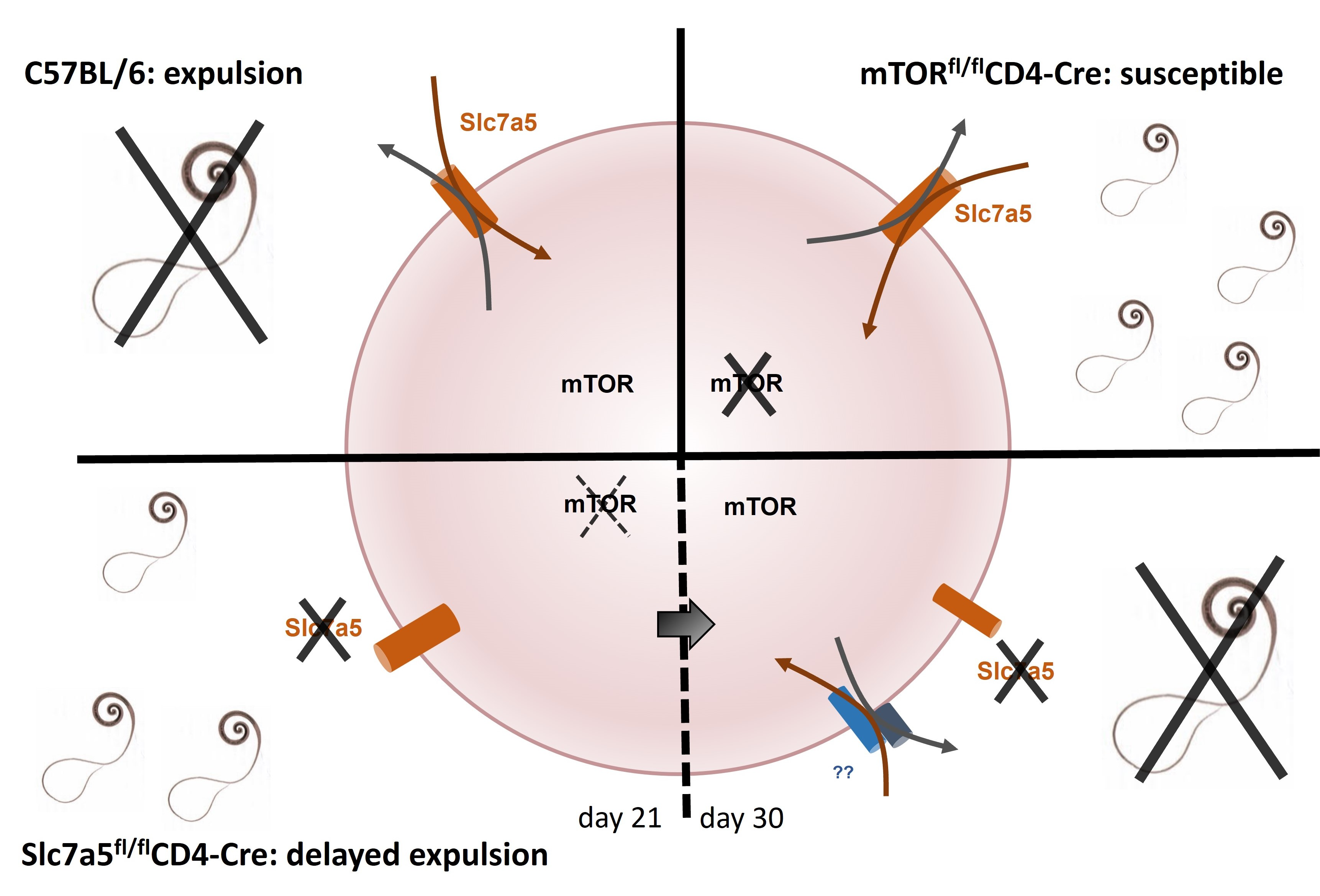
**

**Supplementary Methods: RNA quantification**

RNA purification

Isolated CD4 T cells were kept in pellet at -80^o^C prior to RNA extraction. RNA was

purified from cells using RNeasy RNA purification mini kit (Qiagen, Netherlands)

following the manufacturer’s instructions. Briefly, cells were lysed by the addition of

600μl of RLT buffer. The lysate was homogenized by passing it through a 19G

needle 5-10 times and 600μl of ethanol were added. The lysate was passed

through a spin column and the flow through discarded after an 8000xg spin for 30

seconds. 700μl of RW1 buffer were added to the column to remove other

biomolecules such as carbohydrates, proteins and fatty acids, so the flow through

was discarded again after centrifugation as previously mentioned. 500μl of buffer

RPE were added and flow through was discarded after centrifugation once more.

The spin column was placed in a new tube and RNA was eluted in 30-50μl of

nuclease-free water. After purification, genomic DNA was digested using Turbo

DNA-free kit (Qiagen, Netherlands). 4μl of Turbo DNA-free buffer were added to

the RNA, together with 1μl of DNase. The mixture was incubated at 37^o^C for 30

min. To inactivate the enzyme, DNase inactivation reagent was added. The

purified-DNA-free RNA was transferred to another Eppendorf tube after

centrifugation of the inactivating agent, which forms a pellet. RNA was kept at -

80^o^C until the RT-PCR was performed.

RT-PCR

RNA concentration was assessed using Nanodrop ND-1000 (Nanodrop). 1μg of

RNA was transferred to PCR strip tubes and nuclease-free water was added to

make a volume of 14.3ul. RNA was heated up to 95^o^C and snap cooled on ice.

RNA was then reverse transcribed using GoScript System (Promega, USA). Each

reaction consisted of 6μl of 5X buffer, 1.5μl of dNTP, 3.6μl of MgCl2, 2.5μl of oligo

deoxythymine (dT), 1.5μl reverse transcriptase (RT) and 0.6μl RNasin (also

Promega). Annealing was achieved by leaving samples at 25^o^C for 5 min, which

was followed by a 1 hour extension step at 42^o^C and inactivation of the RT by

heating up to 70^o^C for 15 min. Temperatures were reached using a thermocycler.

qPCR

qPCR was performed in 96-well plates using Sensi-fast SYBR Hi-ROX kit (Bioline,

UK). Reactions consisted of 2μl of cDNA, 10μl of 2x buffer, 6.4μl of water, 0.8μl of

forward primer and 0.8μl of the reverse primer. The primer sets used were the

following:

Slc7a5 (LAT-1)

Forward: CTGGATCGAGCTGCTCATC

Reverse: GTTCACAGCTGTGAGGAGC

Slc7a8 (LAT-2)

Forward: AAGAAGCCTGACATTCCCCG

Reverse: TGTGTTGCCAGTAGACACCC

β- actin

Forward: TCTTGGGTATGGAATGTGGCA

Reverse: ACAGCACTGTGTTGGCATAGAGGT

For the qPCR, samples were heated to 95^o^C for 2 min and then submitted to 40

cycles of the following temperatures: 95^o^C for 5 min, 60oC for 10 min and 72^o^C for

20 min. A melt-profile analysis was performed after completion of the reaction, all

using StepOne system (Thermo Fisher).
